# Cerebellar Atrophy in Cortical Myoclonic Tremor and Not in Hereditary Essential Tremor—a Voxel-Based Morphometry Study

**DOI:** 10.1007/s12311-015-0734-0

**Published:** 2015-10-30

**Authors:** A. W. G. Buijink, M. Broersma, A. M. M. van der Stouwe, S Sharifi, M. A. J. Tijssen, J. D. Speelman, N. M. Maurits, A. F. van Rootselaar

**Affiliations:** 1 Department of Neurology and Clinical Neurophysiology, Academic Medical Center, University of Amsterdam, Room D2-113, P.O. Box 22660, 1100 DD Amsterdam, The Netherlands; 2 Brain Imaging Center, Academic Medical Center, University of Amsterdam, Amsterdam, The Netherlands; 3 Department of Neurology, University Medical Center Groningen, University of Groningen, Groningen, The Netherlands; 4 Neuroimaging Center, University Medical Center Groningen, University of Groningen, Groningen, The Netherlands

**Keywords:** Essential tremor, MRI, VBM, Volumetry, FCMTE, Cerebellum

## Abstract

Essential tremor (ET) presumably has a cerebellar origin. Imaging studies showed various cerebellar and also cortical structural changes. A number of pathology studies indicated cerebellar Purkinje cell pathology. ET is a heterogeneous disorder, possibly indicating different underlying disease mechanisms. Familial cortical myoclonic tremor with epilepsy (FCMTE), with evident Purkinje cell degeneration, can be an ET mimic. Here, we investigate whole brain and, more specifically, cerebellar morphological changes in hereditary ET, FCMTE, and healthy controls. Anatomical magnetic resonance images were preprocessed using voxel-based morphometry. *Study 1* included voxel-wise comparisons of 36 familial, propranolol-sensitive ET patients, with subgroup analysis on age at onset and head tremor, and 30 healthy controls. *Study 2* included voxel-wise comparisons in another nine ET patients, eight FCMTE patients, and nine healthy controls. *Study 3* compared total cerebellar volume between 45 ET patients, 8 FCTME patients, and 39 controls. In our large sample of selected hereditary ET patients and ET subgroups, no local atrophy was observed compared to healthy controls or FCMTE. In ET patients with head tremor, a volume increase in cortical motor regions was observed. In FCMTE, a decrease in total cerebellar volume and in local cerebellar gray matter was observed compared to healthy controls and ET patients. The current study did not find local atrophy, specifically not in the cerebellum in hereditary ET, contrary to FCMTE. Volume increase of cortical motor areas in ET patients with head tremor might suggest cortical plasticity changes due to continuous involuntary head movements.

**Electronic supplementary material:**

The online version of this article (doi:10.1007/s12311-015-0734-0) contains supplementary material, which is available to authorized users.

## Introduction

Essential tremor (ET) is one of the most common neurological disorders, characterized by a progressive postural and kinetic tremor [1, 2]. Moreover, ET is a heterogeneous disorder; patients differ in the presence of head tremor, family history, and response to medication, possibly indicating different underlying disease mechanisms [3]. It has even been suggested that the presence of head tremor and early versus late disease onset might differentiate between ET subtypes [4, 5].

Clinical, imaging, and pathology findings point to cerebellar dysfunction in ET. ET patients can show an ataxic gait, eye movement abnormalities, and intention tremor, and symptoms often diminish upon alcohol consumption [6–14]. Functional and metabolic abnormalities have been demonstrated in the cerebellum and brainstem by functional MRI (fMRI), magnetic resonance spectroscopy, and diffusion tensor imaging [15–18]. Several, mainly structural imaging studies, indicated various cortical changes including volume decrease in the temporal lobe [19–21], frontal lobe [20, 22], parietal lobe [19–22], and occipital lobe [19, 20] (see Sharifi et al.[23] for a review). The jury is still out on whether atrophy is a true hallmark of ET.

Currently, there are three mutually non-exclusive hypotheses about the pathophysiology of ET [24]. Reports of alleviation of tremor after thalamic deep brain stimulation and after stroke within the physiological central motor network, or cerebello-thalamo-cortical network, prompted the hypothesis of essential tremor as an “oscillating network disorder” [25]. A second hypothesis labels ET as a neurodegenerative disorder, with pathology studies showing evidence for structural cerebellar changes, with Purkinje cell loss and axonal swelling, and simultaneous remodeling of the cerebellar cortex [26–31]. A third hypothesis is associating ET with abnormal functioning of the inhibitory neurotransmitter gamma-Aminobutyric acid (GABA).

Another neurological disorder, known to be associated with Purkinje cell changes and cerebellar atrophy, is familial cortical myoclonic tremor and epilepsy (FCMTE), also referred to as familial adult myoclonic epilepsy (FAME). FCMTE is a heritable disease characterized by progressive myoclonus of the distal limbs and infrequent epileptic seizures and signs of cortical hyperexcitability [32–34]. Autosomal dominant FCMTE/FAME has been linked to various chromosomal loci [35–38]. FCMTE has been associated with cerebellar atrophy and decreased cerebellar fiber density [33, 34, 39]. Clinically, the tremulous movements of FCMTE can be confused with ET [40]. It would be of interest to see whether structural cerebellar abnormalities in ET, if present, are comparable to those in FCMTE, considering that Purkinje cells are hypothesized to be affected in both conditions.

This study has been divided in three parts. *Study 1* aims to answer the question whether atrophy is a true hallmark of (subgroups of) ET. In order to accomplish this, we set out to investigate volumetric differences in a large, clinically well-defined, group of hereditary ET patients, compared with healthy controls. In light of the large clinical heterogeneity of ET, for *study 1*, we have selected ET patients with a positive family history and a disease onset before the age of 65. Previously, this operational definition was termed “hereditary ET” [4]. Furthermore, patients had to report a positive effect for propranolol treatment. As it has been suggested that the presence of head tremor and disease onset represent different ET subtypes [4, 5], subgroup analyses were performed in ET patients (1) with and without head tremor and (2) with early versus late onset tremor (before or after the 40 years of age) [41]. For study 1, we expect volumetric changes in the ET group, if present at all, to be confined to the cerebellum.

*Study 2* aims to compare volumetric changes in ET, FCMTE, and healthy controls. We expect no or localized cerebellar atrophy in ET and generalized cerebellar atrophy in FCMTE. To further increase the sensitivity of our data analysis, the spatially unbiased infratentorial template (SUIT [42]) is used, developed specifically for the cerebellum and presently the most accurate method to detect volumetric differences in the cerebellum [42]. Finally, in *study 3*, global cerebellar volume will be compared between ET and FCMTE patients, and healthy controls.

## Materials and Methods

The study was conducted in two academic hospitals in the Netherlands: the Academic Medical Center in Amsterdam (AMC) and the University Medical Center Groningen (UMCG).

### Study 1—ET Patients and Controls

Thirty-six propranolol-sensitive ET patients with familial upper limb tremor and 30 age- and gender-matched healthy controls were included for *study 1*. All subjects were right-handed according to the Annett handedness questionnaire and gave written informed consent before participation. Patients were included when they fulfilled the clinical criteria defined by the Tremor Investigation Group [43], reported a positive effect of propranolol on tremor, had a positive family history of at least one affected relative in immediate family and tremor onset before the age of 65 years, a disease duration longer than 5 years, and were aged 18 years or older. Tremor on and off propranolol medication was recorded on video using the Fahn-Tolosa-Marin Tremor Rating Scale (TRS) parts A and B [44] and assessed, blinded for medication condition, by a movement disorder specialist (JDS). Part A consists of assessment of tremor amplitude during rest, posture, movement, and finger-to-nose manoeuvres. Part B consists of tremor-inducing tasks, including writing, two standardized Archimedes spirals, a line drawing task, and a water pouring task. To determine the treatment effect of propranolol, patients quit their medication minimally 3 days before the off-medication tremor assessment. Exclusion criteria for both groups were (other) neurological disorders and cognitive dysfunction (i.e., mini-mental state examination <26). Furthermore, ET patients using other tremor medication such as anti-epileptic drugs were not included. See Table 1 for full subject characteristics. The study was approved by the medical ethical committees of both centers and conducted according to the Declaration of Helsinki (Seoul, 2008).

**Table 1:** Clinical and demographic characteristics of subjects in studies 1 and 2

Study 1	Controls	ET	
Number	30	36	
M/F	19/11	23/13	
Age (years)	54 ± 15	56 ± 14	
Disease duration (years)	–	27 ± 16	
Head tremor (y/n)	–	13/26	
TRS A + B score off medication	–	23 ± 12	
Propranolol dose (mg)	–	72 ± 64	
Propranolol effect on TRS A + B	–	3.34 ± 3.8	
Gray matter volume (ml)	575 ± 62	573 ± 74	
Study 2	Controls	ET	FCMTE
Number	9	9	8
M/F	6/3	6/3	5/3
Age (years)	43 ± 12	50 ± 18	41 ± 13
Disease duration (years)	–	30.2 ± 18	17 ± 10
UMRS score	–	–	10 ± 10
Gray matter volume	526 ± 70	462 ± 52	435 ± 23

Mean ± standard deviation. Propranolol effect is improvement determined by difference between TRS A + B on and off propranolol medication

*TRS* Tremor Rating Scale

### Study 2—FCMTE Patients, ET Patients, and Controls

We have included nine additional ET patients, matched to eight FCMTE patients, and nine healthy controls for *study 2*, who have been scanned and described in previous reports [39, 45]. See Table 1 for full subject characteristics. ET patients were included when they fulfilled the clinical criteria defined by the Tremor Investigation Group [43]; propranolol responsiveness and a positive family history were not required for *study 2*. ET patients had a moderately severe tremor assessed clinically, but no video recordings are available to assess the Fahn-Tolosa-Marin Tremor Rating Scale. Myoclonus severity for the FCMTE patients was scored using the Unified Myoclonus Rating Scale (UMRS [46]).

### Study 3—Total Cerebellar Volume in FCMTE Patients, ET Patients, and Controls

To assess global cerebellar volume differences, subjects from studies 1 and 2 were pooled.

### Data Acquisition

For *study 1*, a high-resolution anatomical T1 3D Turbo Field Echo (TFE) image was obtained (echo time 3.53 ms, repetition time 9 ms, flip angle 8°, field of view 256 × 256 mm, voxel size 1 mm^3^, number of slices 170. For *study 2*, high-resolution anatomical T1 3D Fast Field Echo (FFE) images were obtained with the same spatial resolution (for details, see van Rootselaar et al. [45]). Foam padding was used to minimize head motion during scanning. Due to differences in acquisition parameters, T1 images of *study 1* and *study 2* could not be pooled for the voxel-based morphometry (VBM) analysis and are therefore analyzed separately. Note that for this reason, results can also not be compared between studies 1 and 2 directly.

### DARTEL Preprocessing (Cerebral Cortex)

Preprocessing and data analysis was carried out with Statistical Parametric Mapping 8 (SPM8, Wellcome Trust Centre for Neuroimaging, UCL, London, UK; http://www.fil.ion.ucl.ac.uk/spm) implemented in MATLAB (Mathworks, Sherborn, MA), using the VBM8 toolbox (http://dbm.neuro. uni-jena.de/vbm/). T1 images were segmented in gray matter and white matter and subsequently spatially normalized using the diffeomorphic anatomical registration through an exponentiated Lie algebra (DARTEL) approach [47]. The resulting transformations were applied to the T1 gray matter segmented images and smoothed with an 8-mm full-width at half-maximum isotropic Gaussian kernel.

### SUIT Preprocessing (Cerebellum)

T1 images were additionally spatially normalized using the spatially unbiased infratentorial template procedure (SUIT version 2.7 [42]). SUIT normalization is known to have more accurate inter-subject alignment of cerebellar structures; therefore, we used an isotropic Gaussian smoothing kernel of 4 mm. The SUIT procedure isolates the cerebellum and brainstem and creates a mask. These masks were manually corrected with the help of MRIcroN (http://www.mccauslandcenter.sc.edu/mricro/mricron). After preprocessing, smoothed modulated normalized data with a voxel size of 1 mm^3^ for DARTEL (181 × 217 × 181 voxels) and SUIT (141 × 95 × 87 voxels) were obtained.

### Obtaining Total Cerebellar Gray Matter Volumes

Masks were back-projected from SUIT space into native subject space using the inverted deformation from standard space to subject space derived from the SUIT normalization procedure. Total cerebellar gray matter volumes were subsequently obtained with the get_totals Matlab function (http://www.cs.ucl.ac.uk/staff/g.ridgway/vbm/get_totals.m).

### Statistical Analysis

Voxel-wise comparisons of the local concentration of gray matter between groups were performed by including smoothed gray matter volumes into a general linear model. All comparisons were corrected for age and total gray matter volume, obtained from the VBM8 procedure described elsewhere (http://dbm.neuro.uni-jena.de/vbm8/ [48]).

For *study 1*, two-sample *t* tests were performed to evaluate local volumetric changes between (1) patients and controls, (2) early onset (<40 years) versus late onset (≥40 years) tremor patients, and (3) head tremor versus no head tremor patients. Multiple regression analyses were performed in the patient group, to correlate voxel-wise, local concentration of gray matter with tremor severity (TRS part A and B), response to propranolol (difference in TRS part A + B between on and off medication), and disease duration.

For *study 2*, local gray matter volume of a separate group of ET patients, FCMTE patients, and healthy controls was compared using a general linear model, by a one-way ANCOVA and post hoc two-sample *t* tests. All contrasts were obtained for the cerebral cortex and cerebellar template. Cluster-wise inference was used (*P* < 0.05 (FWE corrected), cluster-forming threshold *P* < 0.001). The probabilistic atlas of the cerebellar cortex and the Anatomy toolbox were used to determine anatomical locations of volumetric differences [49, 50].

For *study 3*, group differences in total cerebellar volume (TCV) were compared using a one-way ANCOVA, corrected for age and T1 acquisition (*study 1* T1 3D TFE image, *study 2* T1 3D FFE image), and post hoc independent two-sided *t* tests.

## Results

### Subject Characteristics

Table 1 shows clinical and demographic data of the included subjects. Of 36 ET patients included in *study 1*, 13 patients had a head tremor. Mean disease duration did not differ between patients with or without head tremor (head tremor 25.9 years (SD 20.4 years) versus no head tremor 27.6 years (12.9 years)).

### Study 1—ET (Subgroups) Versus Controls

**Fig. 1:**
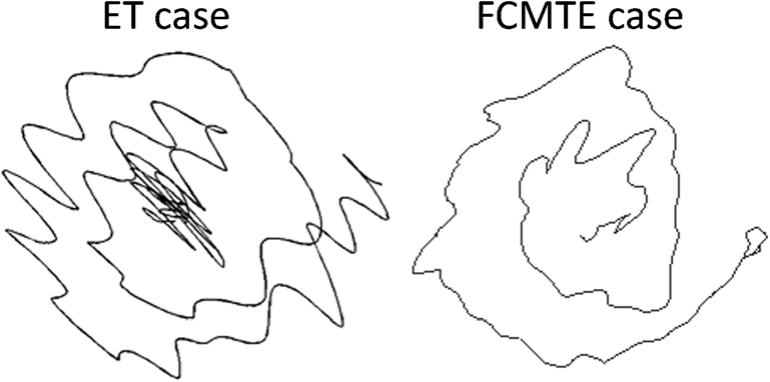
Spiral drawings from an ET and FCMTE patient. Spirals drawn with the right hand by an ET and an FCMTE patient

**Fig. 2:**
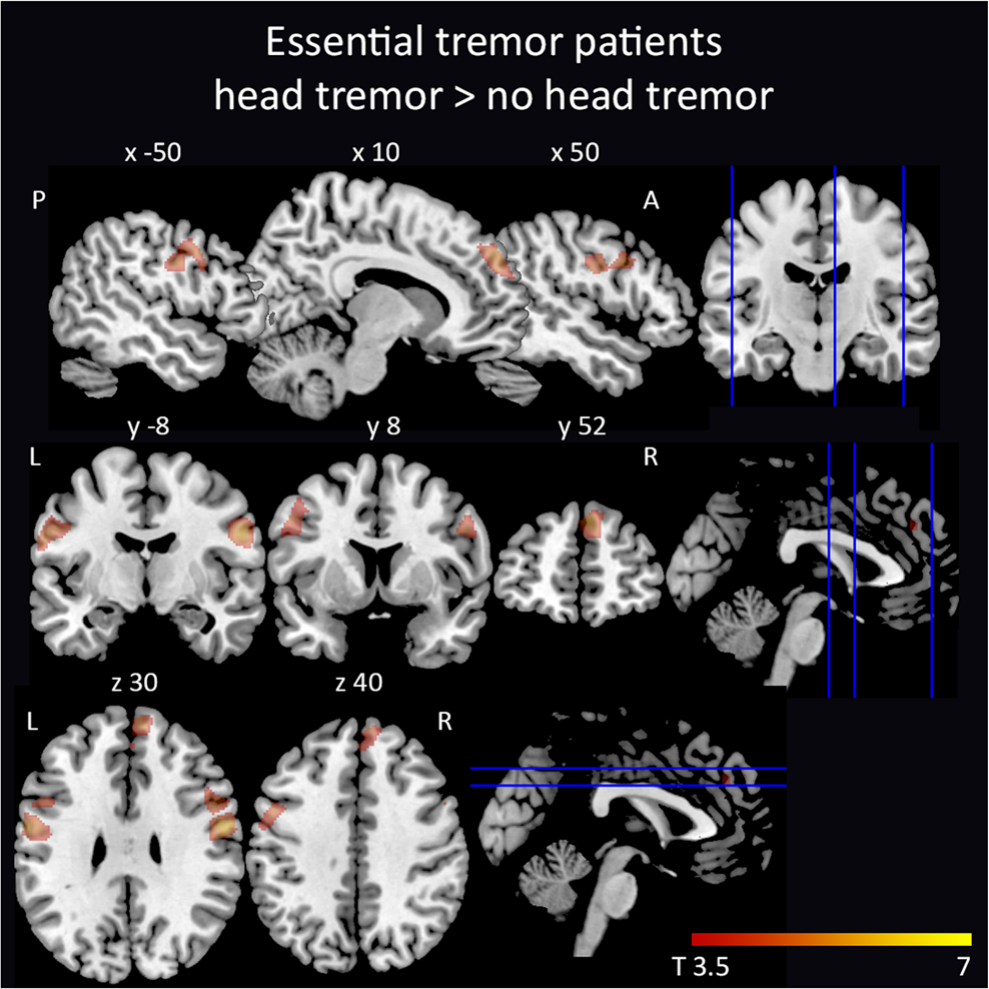
*Study 1*—cortical volume increase in ET patients with head tremor. Increased volume in ET patients with head tremor compared to ET patients without head tremor. Increased volume is mainly confined to the bilateral precentral gyrus and right superior medial gyrus. Cluster-wise inference (*P*< 0.05 (FWE corrected), cluster-forming threshold *P* < 0.001). Results are projected on the ch2better template in sagittal, coronal, and axial views (MRIcroN, http://www.mccauslandcenter.sc.edu/mricro/mricron)

Whole brain and cerebellar VBM analyses did not reveal significant differences between ET patients and controls. Within ET patients, a subgroup comparison between ET patients with and without head tremor showed a significant volume increase in the bilateral precentral and postcentral gyrus and the left superior medial gyrus in ET patients with head tremor (Figs. 1 and 2, Table 2). There was no relationship at whole brain or cerebellar level between an increase and decrease of local gray matter and tremor scores, disease duration, response to propranolol, or early versus late disease onset.

**Table 2:** Local maxima of increased volume in ET patients with head tremor compared to ET patients without head tremor and local maxima of reduced volume in FCMTE compared to ET and controls

Region	Hemisphere	*t* Value	*P*_FWE-corr_	Cluster size	*x*, *y*, *z* in mm		
Study 1—ET head tremor > ET no head tremor							
Postcentral gyrus	Right	6.07	0.002	1396	59	−4	28
Precentral gyrus	Right	5.16			44	17	34
Precentral gyrus	Right	4.96			51	11	33
Superior medial gyrus	Right	5.43	0.016	857	6	54	36
Superior medial gyrus	Right	4.08			2	42	33
Superior medial gyrus	Right	3.99			3	42	42
Postcentral gyrus	Left	5.39	0.001	1414	−60	−3	28
Precentral gyrus	Left	4.74			−50	6	39
Precentral gyrus	Left	4.44			−48	11	31
Study 2—ET > FCMTE							
Cerebellum crus II	Left	5.37	0.005	1160	−25	−77	−42
Cerebellum lobule VI	Left	4.75			−23	−62	−31
Cerebellum crus I	Left	4.53			−34	−50	−35
Study 2—controls > FCMTE							
Cerebellum lobule VIIIa	Right	6.11	<0.001	4522	12	−67	−42
Cerebellum crus I	Right	5.41			25	−74	−34
Cerebellum lobule V	Right	5.33			14	−51	−18
Cerebellum crus I	Left	5.45	<0.001	3188	−20	−74	−33
Cerebellum lobule VI	Left	4.91			−18	−64	−27
Cerebellum crus I	Left	4.70			−37	−49	−36
Cerebellum lobule IX	Left	5.30	<0.001	3133	−14	−46	−48
Cerebellum lobule VIIIb	Left	4.97			−16	−60	−47
Cerebellum lobule X	Left	4.59			−21	−43	−45
Cerebellum lobule IX	Right	5.27	0.01	990	8	−55	−52
Cerebellum lobule VIIIb	Right	4.92			5	−62	−39
Cerebellum lobule IX	Right	4.47			6	−49	−43

Stereotactic coordinates of the local maxima of clusters showing volume reduction in FCMTE compared to ET and controls (cluster-wise inference was used (*P* < 0.05 (FWE corrected), cluster-forming threshold *P* < 0.001). Cluster size is given in number of voxels. MNI stereotactic coordinates for cortical regions, SUIT coordinates for infratentorial regions

### Study 2—Local Cerebellar Changes, FCMTE Versus ET and Controls

**Fig. 3:**
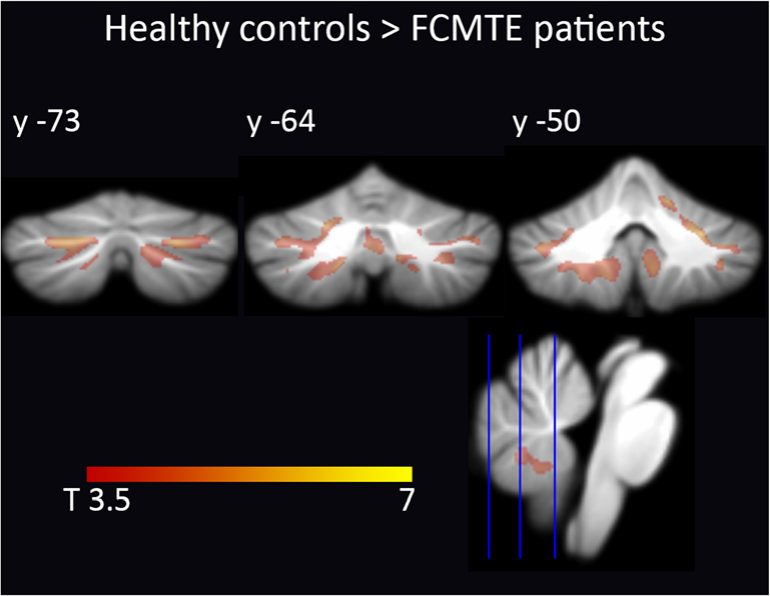
*Study 2*—cerebellar volume reduction in FCMTE. Cerebellar VBM results show volume reduction in FCMTE compared to healthy controls and ET patients. Cluster-wise inference (*P* < 0.05 (FWE corrected), cluster-forming threshold *P* < 0.001). Results are projected on the SUIT atlas [42]

In FCMTE patients compared to a separate group of ET patients and healthy controls, the SUIT analysis revealed widespread gray matter loss throughout the cerebellum, mainly confined to cerebellar motor areas, crus I, and lobules IX and X (Fig. 3, Table 2). Whole brain ANCOVA analysis did not reveal significant differences between ET patients, FCMTE patients, and healthy controls.

### Study 3—Total Cerebellar Volume, Differences Between Pooled Groups

**Fig. 4:**
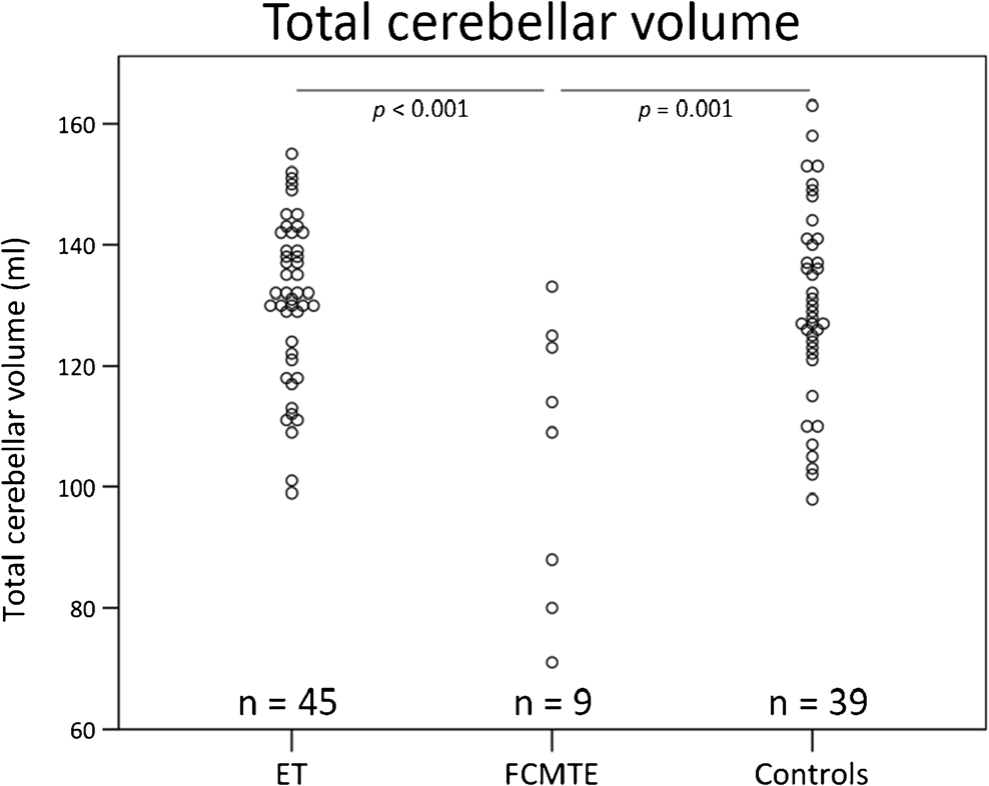
*Study 3*—total cerebellar volume. Total cerebellar volume of combined subjects from studies 1 and 2. *Dot plots* of total cerebellar volume for ET patients, FCMTE patients, and controls, respectively. *P* values based on two-sided *t* tests

ET patients showed a mean TCV of 131 ml (SD 14 ml), for FCMTE patients 105 ml (SD 23 ml), and for controls 130 ml (SD 16 ml). There was a significant group effect for total cerebellar volume (*F*(2) = 6.8, *P =* 0.002, corrected for age and T1 acquisition protocol, Fig. 4). Post hoc independent *t* tests revealed significantly decreased TCV in FCMTE patients compared to controls (*t*[45] = 3.66, *P* = 0.001) and ET patients (*t*[51] = 4.4, *P* < 0.001).

## Discussion

In this report of a large sample of hereditary, propranolol-sensitive ET patients with an age at onset before 65 years, there was no decrease in local cortical or cerebellar gray matter volume compared to age-matched healthy controls. In a subgroup of ET patients with head tremor, we report a volume increase in cortical regions (*study 1*). We report for the first time global and localized cerebellar gray matter reduction in FCMTE, compared to ET patients and healthy controls (*study 2 and 3*).

### Study 1: No Decrease in Cerebellar or Cerebral Volume in Essential Tremor

ET has been associated with Purkinje cell changes [26–29, 32–34, 51]. Our results indicate that the possible Purkinje cell loss in hereditary ET does not give rise to macroscopic atrophy. A previous study by Daniels and colleagues could not objectify cerebellar atrophy in ET compared to controls either; however, the presence of more subtle changes in the cerebellar gray matter could not be excluded [19]. We have focused our analysis on the cerebellum using the SUIT toolbox. The SUIT toolbox provides two advantages compared to the standard analysis. Normalization using the SUIT approach improves the overlap of cerebellar structures between subjects, and by masking the image before reslicing it into atlas space, no supra-tentorial gray matter can bias the results [42]. This allows to state with more certainty that volumetric differences are within the range of age-related atrophy, at least in hereditary ET.

Cortical volumetric changes in ET found in previous studies are not consistent (Supplementary Table 1). The large discrepancies between results of structural imaging studies in essential tremor may be explained by disease heterogeneity, different inclusion criteria and different definitions of ET subgroups between studies and/or methodological differences including differences in magnetic field strength, and use of more liberal statistical thresholds. In fact, results due to methodological or biological differences can appear very similar using volumetric analyses [52]. This makes the interpretation and generalizability of these results challenging. In our analysis, we have chosen for a validated approach with a statistical threshold corrected for multiple comparisons. Based on our results of no volumetric differences related to hereditary ET, combined with previous findings, we postulate that macroscopic volumetric changes are not a characteristic of hereditary ET, at least not perceptible with a smoothing kernel of 4 mm [19].

As mentioned in the Introduction, there are three mutually non-exclusive hypotheses regarding the pathophysiology of ET. The first being that of an “oscillating network disorder,” the second hypothesis regards ET as a neurodegenerative disorder, with evidence for Purkinje cell loss and axonal swelling [26–31]. Finally, ET is associated with abnormal functioning of the inhibitory neurotransmitter GABA. With respect to the neurodegenerative hypothesis, one could hypothesize that Purkinje cell loss should give rise to macroscopic cerebellar atrophy, as is observed in FCMTE patients [33, 34, 53]. However, most pathology studies in ET emphasize altered Purkinje cell *morphometry* instead of large decrease in Purkinje cell *count* [54], as opposed to the more extended Purkinje cell loss in FCMTE [33, 34, 53]. GABAergic neurotransmission dysfunction within the cerebellum has been reported in ET, with increased ^11^C-flunazenil binding to GABA receptors in the cerebellar cortex, increasing with tremor severity, and in the dentate nucleus, suggesting functional cerebellar changes [55, 56]. Additionally, a decrease in GABA receptors has been observed in the dentate nucleus in ET [57]. Our findings do not refute either hypothesis but are, because of lack of local atrophy, to our opinion consistent with functional cerebellar changes in ET.

### Volumetric Changes Related to Head Tremor

We did observe increased volume in cortical regions related to motor control in ET patients with head tremor compared to ET patients without head tremor. This increase in volume might be explained in multiple ways. The most plausible hypothesis is that the volumetric increase of cortical motor regions indicates that cortical plasticity occurs in response to continuous involuntary head movements. If this was to be true, one could speculate that a volume increase of the motor cortex in general would be associated with ET, since the hands are well-represented in the human motor cortex. One assumption could be that limb tremor is possibly less omnipresent throughout the day compared to head tremor and therefore does not give a volume increase of the associated hand areas. Alternative explanations might be that these areas are primarily affected in the ET subtype exhibiting head tremor or that these patients are more prone to develop head tremor to start with. In this light, it would be of interest to compare ET patients with head tremor with patients with cervical dystonia, to observe whether similar plastic changes occur. Gray matter volume increase in the primary motor cortex has been observed previously in patients with dystonia and even more clearly in patients with cervical dystonia [58].

Two previous studies, from the same group, reported cerebellar vermal gray matter reduction in ET patients with head tremor [5, 59]. We were unable to confirm these results, even in a post hoc analysis with a statistical threshold of *P* = 0.001, uncorrected for multiple comparisons, to further increase the specificity of our findings. In previous studies, the head tremor group was significantly older, with a mean age difference of 10 years with ET patients without head tremor [5]. In our study, mean age did not differ between ET patients with and without head tremor. Our study was not specifically powered to assess gray matter reduction related to head tremor in ET. Therefore, another explanation could be an insufficient sample size of subgroups (13 patients in the current study versus 19 and 20 patients with head tremor in the previous studies) [5, 59].

### Study 2 and 3: Cerebellar Atrophy in FCMTE

This is the first report of widespread regional cerebellar atrophy in FCMTE compared to ET and healthy controls. Previously, pathology studies revealed global cerebral and cerebellar atrophy in FCMTE [33, 34]. Widespread volumetric changes throughout the cerebellum could be caused by the evident Purkinje cell loss, observed in FCMTE [32–34, 39]. Cerebellar fiber density does appear to be decreased in FCMTE compared to ET and healthy controls [39]. In this same study, no decrease in cerebellar volume was observed in FCMTE. However, in this study, the authors used a less sensitive technique by annotating the cerebellum based on the fractional anisotropy and mean diffusivity volumes, with a lower spatial resolution [39]. Functional MRI and (1)H-MR spectroscopy studies have provided evidence for a crucial role of the cerebellum in the pathophysiology of FCMTE [45, 60] that were confirmed with PA studies showing almost isolated Purkinje cell changes [33, 34]. In FCMTE, tremulous movements are believed to originate from the sensorimotor cortex, in fact being cortical myoclonus. Cerebellar pathological changes, reflected in our finding of marked cerebellar atrophy, can lead to decreased cerebellar inhibition of the dentato-thalamic-cortical tracts [33, 34, 61, 62]. It is hypothesized that reduced inhibitory Purkinje cell output onto the dentate nucleus causes this decreased inhibition [61, 62].

In conclusion, based on the current study, atrophy seems not to be a characteristic of hereditary ET. Considering that our study has failed to find local volumetric changes in a selected group of hereditary ET patients indicates that even if these changes were to be present, they would be smaller than age-related differences. We furthermore have shown a volumetric increase of cortical motor areas related to head tremor. Moreover, in a clear Purkinjopathy, such as FCMTE, widespread cerebellar volumetric changes were objectified.

## Electronic supplementary material

Below is the link to the electronic supplementary material.

Supplementary Table 1 (DOC 56 kb)
