# Supplementary material for: Cerebellar Atrophy in Cortical Myoclonic Tremor and Not in Hereditary Essential Tremor—a Voxel-Based Morphometry Study
Source: Cerebellum. 2015 Oct 30;15(6):696–704. doi: 10.1007/s12311-015-0734-0 (PMC5097101; doi:10.1007/s12311-015-0734-0)
Supplement: Supplementary file 1 — (DOC 56 kb) [file 12311_2015_734_MOESM1_ESM.doc]

*Supplementary table 1. Previous volumetric studies in essential tremor.*

| Reference | No. of patients/controls | Field strength | Data analysis | Findings |
| --- | --- | --- | --- | --- |
| Daniels et al., 2006 [1] | 14 ET-PT/ 14 HC  13 ET-IT/ 13 HC | 1.5 T | WB GM, WM,  small volume correction for cerebellum (p corr voxel level < 0.05). Smoothing unknown. | ET-IT: ↑ GM volume temporoparietal junction, unilateral occipital cortex |
| Quattrone et al., 2008 [2] | 30 ET-a/ 20 ET-h | 1.5 T | WB GM + cerebellar volume. (p corr cluster level < 0.001). 10mm smoothing. | h-ET: ↓ GM cerebellar vermis and cerebellar volume |
| Cerasa et al., 2014 [3] | 30 ET-a/ 20 ET-h | 1.5 T | FreeSurfer (p uncor < 0.05) | h-ET: ↓ cerebellar volume |
| Benito-León et al., 2009 [4] | 19 ET/ 20 HC | 3T | WB GM, WM (p uncorr < 0.001). 6mm smoothing. | Widespread cortical and cerebellar differences (↓or↑) |
| Bagepally et al., 2012 [5] | 10 ET/ 10 ET-h/17 HC | 3T | WB GM, WM, ICV. (p uncorr < 0.001). 8mm smoothing. | Widespread cortical and cerebellar atrophy |
| Lin et al., 2013 [6] | 10 ET/10 PD/ 13 HC | 3T | WB GM  (p uncorr < 0.05). 8mm smoothing. | ↓ caudate, middle temporal pole, insula, precuneus, ↑ middle temporal gyrus, precentral gyrus |

The presence of cortical and cerebellar atrophy in ET remains unclear, partly because of heterogeneous results, varying methods, statistical thresholds and patient characteristics. ET essential tremor; PT postural tremor; IT intention tremor; HC healthy controls; ET-a only arm tremor; ET-h head and arm tremor; PD Parkinson’s disease; WB whole brain; GM gray matter; WM white matter; corr corrected for multiple comparisons; uncorr uncorrected for multiple comparisons; T Tesla.

[1] Daniels C, Peller M, Wolff S, Alfke K, Witt K, Gaser C, et al. Voxel-based morphometry shows no decreases in cerebellar gray matter volume in essential tremor. Neurology 2006;67:1452–6.

[2] Quattrone A, Cerasa A, Messina D, Nicoletti G, Hagberg GE, Lemieux L, et al. Essential head tremor is associated with cerebellar vermis atrophy: a volumetric and voxel-based morphometry MR imaging study. AJNR Am J Neuroradiol 2008;29:1692–7.

[3] Cerasa A, Nisticò R, Salsone M, Bono F, Salvino D, Morelli M, et al. Neuroanatomical correlates of dystonic tremor: a cross-sectional study. Parkinsonism Relat Disord 2014;20:314–7.

[4] Benito-León J, Alvarez-Linera J, Hernández-Tamames JA, Alonso-Navarro H, Jiménez-Jiménez FJ, Louis ED. Brain structural changes in essential tremor: voxel-based morphometry at 3-Tesla. J Neurol Sci 2009;287:138–42.

[5] Bagepally BS, Bhatt MD, Chandran V, Saini J, Bharath RD, Vasudev MK, et al. Decrease in cerebral and cerebellar gray matter in essential tremor: a voxel-based morphometric analysis under 3T MRI. J Neuroimaging 2012;22:275–8.

[6] Lin C-H, Chen C-M, Lu M-K, Tsai C-H, Chiou J-C, Liao J-R, et al. VBM Reveals Brain Volume Differences between Parkinson’s Disease and Essential Tremor Patients. Front Hum Neurosci 2013;7:247.
